# Unpacking the role of school climate factors in fostering teacher enjoyment and self-efficacy in higher education

**DOI:** 10.1371/journal.pone.0329001

**Published:** 2025-08-13

**Authors:** Xiaoyi Bing, Jianqiao Cai

**Affiliations:** School of Educational Science, Hunan Normal University, Changsha, China; University of Tartu, ESTONIA

## Abstract

The enjoyment and self-efficacy of teachers warrant significant attention, particularly given the global prevalence of high attrition and turnover rates in the profession. This study investigates the interrelationships among school climate factors, teacher self-efficacy, and enjoyment in higher education settings. Specifically, it aims to (a) examine how teachers perceived school environment factors influence their self-efficacy and enjoyment and (b) determine whether teachers’ self-efficacy mediates the effect of perceived school climate factors on teacher enjoyment. In so doing, three instruments including Perceived School Climate Scale (R-SLEQ), Teacher Self-Efficacy Scale (TSES), and Teacher Enjoyment Scale (TES) were used. Employing snowball sampling, we recruited 791 college teachers from 24 provinces in China. Structural equation modeling revealed that specific school climate factors significantly contribute to both teachers’ enjoyment and self-efficacy. Notably, colleague collaboration, resource availability, participative decision-making, and instructional innovation demonstrated direct positive effects on teacher enjoyment. Instructional innovation and colleague collaboration further exerted indirect effects on enjoyment through the mediation of self-efficacy. Finally, the practical implications of these findings for teacher education are discussed.

## Introduction

Teachers experience a wide range of distinct emotions, varying in intensity while engaging in instruction and interacting with students [1]. These emotions have a salient impact on how well educational systems function [2]. While extensive research has examined negative affective states in teachers—including anger, burnout, and stress [3–7], research on positive teacher emotions remains relatively scarce [8,9]. This study focused on one key manifestation of positive emotion in teachers: enjoyment, given that educators’ positive emotions like enjoyment, happiness, and enthusiasm often arise from similar emotional triggers—particularly student engagement, good behavior, and learning motivation [10].

Teachers are expected to be joyful during teaching, as their enjoyment not only enhances student learning [11] but also reduces teacher burnout and promotes higher teaching quality [12–14]. While several studies have explored the antecedents of teaching enjoyment [15,16], critical gaps persist in our understanding. Notably, the relationship between organizational factors and teacher enjoyment is not well understood, with most research focusing on enjoyment within the classroom context. Furthermore, while studies have shown a positive relationship between school climate and teacher enjoyment [17,18], there is a lack of research examining how specific aspects of school climate contribute to teacher enjoyment, or if certain factors hold more significance. Similarly, teacher self-efficacy—a key concept in the teaching domain—has been shown to correlate with school climate [19,20], yet its relationship with specific school climate factors remains underexplored. To fill these gaps, the present research sought to explore the hypothesis that teachers’ enjoyment and self-efficacy are positively associated with school climate factors, considering the ongoing interactions between teachers and the various environmental systems they are part of [21]. Additionally, drawing on prior studies in instructional research, we further propose that teacher self-efficacy acts as a key mechanism linking school climate factors to teacher enjoyment, given that school climate shapes teacher self-efficacy while teachers’ emotional experiences stem from both the surrounding environment and psychological sources [22,23].

Accordingly, the study seeks to address the following research questions: (1) How do school climate factors directly influence teacher enjoyment in Chinese college settings? (2) How do school climate factors directly impact teacher self-efficacy in this context? (3) Do school climate factors indirectly influence teacher enjoyment through the mediation of teacher self-efficacy?

## Literature review

### Teacher enjoyment

Consistent with prior research on teacher professional development, this study examines enjoyment as a key indicator of teachers’ positive emotions. Enjoyment was identified as a prominent and commonly observed positive emotion experienced by teachers, as noted in studies by Sutton and Wheatley, Burić et al., and Keller et al. [1,9,24]. It is described as an individual’s sense of happiness and satisfaction arising from teaching and interacting with students [24], or the enthusiasm felt while teaching, as described by Keller and colleagues [25].

Research demonstrates that teacher enjoyment yields significant benefits across multiple dimensions. At the student level, it enhances students’ perceptions of teaching quality [16,26], improves teacher-student relationship quality [8,27], and promotes student outcomes including learning enjoyment [11], motivation [28], engagement [10], and academic achievement [29]. For teachers themselves, enjoyment is associated with enhanced job satisfaction [6,30–32], teaching motivation [33], well-being [34], health [35,36], and instructional behavior [8,37]. Frenzel identified three sources of enjoyment: anticipatory joy from an upcoming event, enjoyment derived from engaging in a pleasurable activity, or satisfaction and happiness stemming from a previous desired event or result [8]. In the context of teachers, teachers’ self-efficacy and engagement emerge as significant predictors of their positive emotions, including enjoyment [14,26,38–40], though comparative evidence suggests student engagement exerts stronger influences on teachers’ emotions than self-efficacy [27]. Furthermore, Frenzel et al. identified a reciprocal relationship between teachers’ and students’ enjoyment [11], where students’ initial learning enjoyment enhances teachers’ perceived classroom engagement, thereby reinforcing teachers’ positive affect [8,41]. Diary studies demonstrated that students’ motivation and discipline significantly predict teachers’ enjoyment [15]. In addition, Taxer et al. found that positive teacher-student relationships protect against emotional exhaustion in teachers by sustaining the level of enjoyment they feel in the classroom [42]. Outside classroom, research has shown that teachers with a positive view of their work environment experience greater enjoyment in teaching [17]. Similarly, Sutton emphasized the relevance of teachers’ positive emotions to their everyday work [43].

### Teacher self-efficacy

Teacher self-efficacy, as described in Bandura’s social cognitive theory [44], refers to a teacher’s belief in their capability to successfully foster student engagement and facilitate learning, even with challenging or unmotivated students [39]. This complex construct encompasses teachers’ evaluations of their own chances of succeeding in managing the classroom, engaging students, and implementing teaching strategies [39,45,46].

Recent studies have empirically linked various factors to teacher self-efficacy. Research has shown that school-related factors significantly impact teachers’ self-efficacy [47–49], including school resources and support, teacher perceived collegial leadership, and a positive atmosphere among school staff (i.e., teacher affiliation), all of which are key drivers [50–53]. Conversely, occupational stressors such as burnout and job-related stress have been shown to decrease teachers’ efficacy beliefs [6,51,54–56]. In turn, teacher self-efficacy positively impacts several important teacher outcomes, including work engagement [57–61], job satisfaction [62–66], teaching effectiveness [67–69], well-being [39,69], and positive emotions such as joy and pride [70,71]. Notably, Tschannen-Moran and Hoy highlighted the significance of teacher self-efficacy in promoting their positive emotions [39]. In line with this, later investigations done by Kunter et al. and Xiao et al. further confirmed the strong relationship between teacher self-efficacy and enjoyment [40,72].

### School climate

The empirical research on school climate began in the 1950s [73], but there is no universal agreement among researchers on its definition [74]. Concentrating on the teachers’ perceptions regarding school climate, this paper adopts a teacher-oriented definition, where school climate is defined as “the psychosocial context in which teachers work and teach” [75]. Scholars and practitioners have recognized various components of school climate and have developed scales to measure it (e.g., Questionnaire on Teacher Interaction (QTI), School-level Environment Questionnaire (SLEQ), and Classroom Environment Questionnaire (ICEQ)) [74].

Extensive evidence demonstrates school climate’s multifaceted influence on educational stakeholders. For students, a supportive school climate is associated with enhanced well-being, increased engagement, improved academic achievement, decreased aggression, fewer suspensions, and other positive outcomes [73,74,76,77]. For teachers, studies have found that a positive school climate is associated with lower stress, increased efficacy, job satisfaction, and teacher retention [4,19,78–81]. In addition, it is widely recognized that a supportive school climate significantly shapes teachers’ perceptions, which in turn can improve student learning outcomes [82]. In examining the relationship between school climate factors and teachers’ self-efficacy, Aldridge and Fraser identified three key school climate factors-adequate resources, approachable and supportive principals, and affiliation-that enhance teacher self-efficacy by fostering a stronger perception of institutional goal alignment [19]. Additionally, other studies have further emphasized the positive relationship between teacher self-efficacy and various school climate elements, such as instructional innovation, a supportive working environment, collegial leadership, teachers’ perceptions of professionalism, affiliation, and staff autonomy [4,20,48,83,84]. Regarding emotional outcomes, research by Zhang and colleagues indicated that teachers’ perception of school climate indirectly influences foreign language teachers’ enjoyment via the mediation of their self-efficacy as well as psychological well-being [17]. Additionally, Taxer and fellow researchers demonstrated that positive teacher-student relationships can help prevent emotional exhaustion and increase teacher enjoyment in the classroom [42].

Taken together, current literature clearly points to the relationships between school climate and teacher self-efficacy, school climate and teacher enjoyment, as well as teacher self-efficacy and enjoyment. However, the specific causal mechanisms underlying the interplay among all three constructs remain underexamined [18], especially given that the concept of teacher enjoyment is still in its early stages of development [17]. Moreover, there is a notable gap in the literature regarding how different aspects of school climate impact both teacher self-efficacy and enjoyment.

### Theoretical framework and hypothesized model

When examining teachers’ enjoyment, it is crucial to acknowledge that their emotions are neither purely internal psychological states nor solely determined by external environments; rather, they emerge from dynamic person-environment transactions [22]. As Liu argued, teacher anxiety, traditionally seen as a psychological factor, is actually an eco-psychological construct [85]. Similarly, to fully understand teachers’ enjoyment, it is imperative to consider not only the internal psychological factors but also the broader ecological influences that affect teachers’ emotional experiences.

Bronfenbrenner’s ecological system theory highlights how individuals develop within complex, interacting social and cultural environments [86]. It views the individual situated environment as a layered and complex system, comprising a micro-system, meso-system, exo-system, and macro-system. In understanding teachers’ emotional experiences, Cross and Hong extended the theory by integrating it with emotion research. They refined and expanded the original framework to explore how different environmental levels interact and influence teacher emotions [87]. Specifically, the micro-system, as the innermost layer, refers to the emotional dynamics between teachers and their immediate social interactions, including students, colleagues, and parents. The mesosystem represents the interactions between different microsystems, which examines how the relationships between teachers and students, teachers and parents, and teachers and colleagues influence teachers’ emotions. The exosystem consists of the broader settings in which teachers are not directly involved but which still influence their emotional experiences. This could include the school administration, teacher-parent organizations, local community organizations, or even government policies related to education. Lastly, the macrosystem includes the overarching cultural, economic, political, and societal structures that shape teachers’ emotional experiences [88–90].

**Fig 1:**
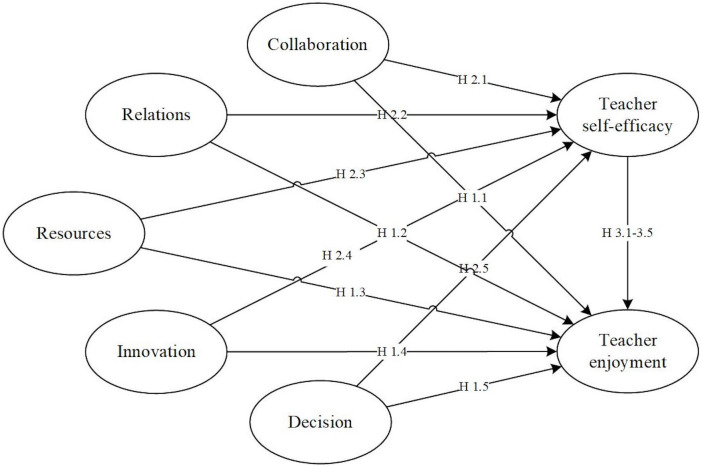
The hypothesized model of the study. Note: H1.1-H1.5 hypothesized the relationships between school climate factors and teacher enjoyment; H2.1-H2.5 hypothesized the relationships between school climate factors and teacher self-efficacy; H3.1-H3.5 hypothesized the mediation effects of teacher self-efficacy on school climate factors and teacher enjoyment.

Following this framework, our research explored teachers’ emotional experiences by examining their interactions across both the microsystem and mesosystem. According to the theory, the microsystem encompasses teachers’ direct, immediate interactions within a specific setting, such as one-on-one exchanges between teachers and students, while the mesosystem captures the linkages between these microsystems, for instance, how teacher collaboration with colleagues can influence classroom practices. At the microsystem level, we focused on teachers’ direct interactions with students and colleagues, examining how these interactions contribute to emotional outcomes (i.e., teacher self-efficacy and enjoyment). At the mesosystem level, we explored how other school climate factors—decision-making, school resources, and instructional innovation—interact to shape teachers’ emotional experiences. While prior studies have touched upon the impact of school climate on both teacher enjoyment and teacher self-efficacy (see above literature), this research systematically delved into the layers of the environment by adopting the micro- and mesosystem from the reconceptualized ecological framework. This approach provided us with a more detailed insight into the interplay among school climate factors, teacher self-efficacy, and their enjoyment in teaching. Drawing from the theoretical foundation and aforementioned body of literature, our study aimed to explore the underlying correlations between the five aspects of school climate, teacher self-efficacy, and teacher enjoyment. Fig 1 illustrates the conceptual model representing our hypotheses.

## Method

Based on our conceptual framework, we used a cross-sectional survey methodology following quantitative approaches. This section presents detailed information regarding the research participants, data gathering process, statistical analysis, main variables, and associated measures.

### Ethics declarations

All methods were carried out in accordance with relevant guidelines and regulations. The experimental protocols were approved by the Ethics Committee of Hunan Normal University (approval number: 2024429). All participants provided electronic informed consent to participate in this research.

### Participants

A total of 791 Chinese college teachers from various provinces participated in the study. They were recruited through a call for participation on social media or via direct invitations. Among the participants, 493 were female (62.3%) and 298 were male (37.7%). The majority of respondents were aged between 26–35 (n = 238; 30.1%) and 36–45 (n = 305; 38.6%). In terms of teaching experience, most participants had been teaching as college teachers for one to 10 years. Regarding professional titles, the largest group consisted of lecturers (n = 333, 42.1%) and associate professors (n = 252, 31.9%). In terms of educational qualifications, 198 teachers held a PhD, 448 held an MA degree, 141 had a BA degree, and four had an associate degree.

### Measurements

This section first outlines the original design of the instruments used, followed by results from confirmatory factor analyses (CFA) to assess their construct validity in the context of our study.

#### Instruments.

To ensure the validity of this study, the first author, holding a master’s degree in Translation and Interpreting, translated and then back-translated all measures employed in this study.

*Perceived school climate scale (R-SLEQ):* We used the Revised School-Level Environment Questionnaire (R-SLEQ), developed by Johnson et al., to measure teachers’ views on their school climate [75]. This instrument consists of 21 items across five dimensions: (a) colleague collaboration, (b) teacher-student relations, (c) school resources, (d) decision-making, and (e) instructional innovation. Participants rated items using a 5-point scale, with 1 denoting “strongly disagree” and 5 denoting “strongly agree”. Among the 21 questions, 8 were negatively phrased and reverse scored, such as “Good teamwork is not emphasized enough at my school.” (1 = strongly agree, 5 = strongly disagree). The R-SLEQ exhibited great reliability, with a reported coefficient of 0.90 that ranged between 0.77 and 0.86 for the five sub-scales [75]. The scale’s robust construct validity was further validated by Hosford and O’Sullivan [91]. In this study, the reliability index of the R-SLEQ, evaluated through Cronbach’s alpha, varied between 0.74 and 0.86.

*Teacher self-efficacy scale (TSES):* To measure teacher self-efficacy, we employed the short form of Teachers’ Sense of Efficacy Scale (TSES) developed by Tschannen-Moran and Hoy [39]. This scale evaluates teachers’ beliefs in their ability to (a) engage students, (b) apply instructional strategies, and (c) manage classroom. The instrument consists of 12 items, rated on a 9-point scale from 1 (nothing) to 9 (a great deal). The TSES demonstrated strong reliability, with reported coefficients for instructional strategies, classroom management, and student engagement of 0.86, 0.86, and 0.81, respectively, accounting for 65% of the variance in teachers’ responses [39]. Klassen et al. further validated the validity of the TSES measure across five different nations [62]. In this study, the internal consistency of the TSES was confirmed with a Cronbach’s alpha of 0.87.

*Teaching enjoyment scale (TES):* To measure teacher enjoyment, the Teacher Emotions Scale (TES) developed by Frenzel and his fellow researchers was used [12]. This scale includes three main emotions: happiness, anxiety, and anger, with a total of 12 items. Our research focused on the sub-scale measuring teachers’ positive emotion—enjoyment in the classroom. The subscale contained four items: “I generally enjoy teaching.”; “I generally have so much fun teaching that I gladly prepare and teach my lessons.”; “I often have reasons to be happy while I teach.”; and “I generally teach with enthusiasm.” Participants rated these items on a 4-point scale from “strongly disagree” to “strongly agree.” In Frenzel et al.’s study, Cronbach’s alphas for the entire TES and for the enjoyment subscale were 0.90 and 0.77, respectively [12]. In our study, the reliability of the TES was confirmed with a Cronbach’s alpha of 0.92.

#### Measurement validation.

To ensure the construct validity, we conducted confirmatory factor analyses (CFA) on each scale individually to test the proposed factor structure of the measurement tools. First, the structure of the R-SLEQ with five constructs (collaboration, student relations, school resources, instructional innovation, and decision-making) was probed for low estimates in standardized estimation. The standardized estimates for individual items ranged from 0.569 to 0.868. Second, the TSES was evaluated, with all twelve items set to load on one factor. The results of the factor loading varied from 0.316 to 0.896. Therefore, six items (CM1, SE1, SE2, SE4, IS2, IS4) were removed due to lower factor loadings (lower than 0.4). Following adjustments, the final construct demonstrated excellent fit indices for the data. Third, for the TES, all four items were loaded onto one factor, with factor loadings ranging from 0.819 to 0.889.

Following adjustments to the individual models, we tested the final CFA model incorporating all measurement scales. The results indicated an excellent fit to the data: CMIN/df = 1.755, RMSEA = 0.035, CFI = 0.968, TLI = 0.964. We then assessed the model’s convergent and discriminant validity for each construct. Analysis in Table 1 revealed that all variables exhibited CR values above 0.7, demonstrating an acceptable reliability. Additionally, the mean variance for each factor exceeded 0.5, supporting the model’s convergent validity. The correlations between all pairs of factors were significant at *p* < .01, signifying large effect sizes. Moreover, the square root of AVE values (bolded in the table) was above the inter-correlations of the factors, confirming discriminant validity, as defined by Fornell and Larcker [92].

**Table 1:** Discriminant validity and composite reliability of the scales.

		1	2	3	4	5	6	7
**R-SLEQ**	Innovation	**.775**						
	Decision	.222**	**.721**					
	Relations	.198**	.19**	**.728**				
	Resources	.224**	.239**	.172**	**.790**			
	Collaboration	.172**	.186**	.149**	.183**	**.732**		
**TSES**	TSE	.359**	.163**	.08**	.183**	.175**	**.772**	
**TES**	TE	.221**	.164**	.084**	.192**	.13**	.407**	**.855**
	**AVE**	.600	.520	.529	.624	.535	.595	.732
	**CR**	0.856	0.761	0.817	0.866	0.872	0.893	0.916

CR = composite reliability; AVE = average variance extracted; TSE = Teacher Self-efficacy; TE = Teacher Enjoyment. **p < .01.

### Data collection

The survey was administered online and included sections for demographic information as well as three scales: R-SLEQ, TSES, and TES. It was distributed via a Weblink and WeChat application, with some teachers and administrators voluntarily sharing the survey link with colleagues and acquaintances, helping to broaden the reach for data collection. Participants’ personal identification information like real name and contact information were not required in the form, and they were all fully informed that their personal information would be safely kept and accessible only to the research team. Additionally, there was no set deadline for survey completion, allowing respondents to answer at their convenience. Detailed instructions were provided for completing the questionnaires, and participants were advised to carefully consider their answers and complete the survey whenever and wherever it was most convenient for them. The data collection phase lasted for two months, from 3^rd^ July to 3^rd^ September 2024, resulting in 791 completed surveys with no missing data.

### Data pre-processing

Prior to analysis, pre-processing steps were undertaken to ensure data quality. The dataset was thoroughly reviewed with the assistance of three attention check questions embedded in the survey, such as, “Please choose Neutral for this question if you are attentively filling out the questionnaire.” and “To answer this question, please choose Agree.” Following these checks, 182 responses were identified as inadequate and excluded from the analysis. This resulted in a final sample size of 609 valid cases.

### Data analyses

Data analysis was performed using SPSS 27.0 and AMOS 26.0. To assess the construct validity of the measurement model, a Confirmatory Factor Analysis (CFA) was conducted with the three scales (see Measurement validation). Subsequently, Structural Equation Modeling (SEM) was employed to examine the structural model and analyze the path relationships between constructs [93]. Furthermore, a bootstrapping procedure consisting of 5000 re-samplings, along with a 95% bias-corrected percentile interval and a percentile confidence interval, was carried out to test the possible mediating effects of teacher self-efficacy.

## Result

The results are presented in three parts. First, we report the descriptive statistics for the study variables. Next, we analyze the relationships among the five dimensions of perceived school climate, teacher enjoyment, and teacher self-efficacy. Finally, we conduct mediation analyses to examine the mediating role of teacher self-efficacy in the relationships.

### Descriptive statistics

The mean scores, standard deviations, skewness, and kurtosis of each latent variable were computed (see Table 2).

**Table 2:** Descriptive statistics of the scores (N = 609).

		Cronbach’s Alpha	Min.	Max.	M	SD	Skewness	Kurtosis
**R-SLEQ**	Collaboration	0.86	1.33	5	3.97	0.70	−1.356	2.087
	Relations	0.82	1.25	5	3.92	0.81	−1.03	1.153
	Resources	0.86	1.25	5	3.86	0.84	−1.031	0.888
	Decision	0.74	1	5	3.93	0.87	−1.052	1.364
	Innovation	0.85	1	5	3.38	1.05	−0.262	−0.895
**TSES**	TSE	0.87	1.33	8.5	3.76	1.27	1.489	2.474
**TES**	TE	0.92	1	4	2.95	0.79	−0.456	−0.534

### Structural model and mediation

Regarding the structural model, we conducted our analysis using AMOS, based on variance-covariance matrices and the maximum likelihood estimation method. The results from the structural equation modeling (SEM) indicated a good fit to the data: CMIN/df = 2.524, RMSEA = 0.05, CFI = 0.934, TLI = 0.928. Table 3 presents the results of the SEM analysis, while Table 4 outlines the mediation effects of teacher self-efficacy.

**Table 3:** SEM results.

			*β*	S.E.	C.R.	*P*
TSE	<---	Collaboration	0.162	0.079	3.679	***
TSE	<---	Relations	−0.039	0.07	−0.871	0.384
TSE	<---	Resources	0.084	0.055	1.95	0.051
TSE	<---	Decision	0.071	0.064	1.561	0.119
TSE	<---	Innovation	0.232	0.046	5.197	***
TE	<---	Collaboration	0.141	0.055	3.225	0.001
TE	<---	Resources	0.166	0.039	3.81	***
TE	<---	Innovation	0.171	0.032	3.884	***
TE	<---	Decision	0.129	0.045	2.792	0.005
TE	<---	Relations	−0.008	0.05	−0.184	0.854

*p < .05, **p < .01, ***p < .001.

**Table 4:** Indirect effects of perceived school climate factors on TE through TSE.

Path	Bootstrapping		95% C.I.
	Est.	Std. Error	
Innovation → TSE → TE	0.079	0.017	[.047,.116]
Decision → TSE → TE	0.022	0.016	[-.011,.053]
Resources → TSE → TE	0.028	0.015	[0.00,.058]
Relations → TSE → TE	−0.012	0.016	[-.044,.020]
Collaboration → TSE → TE	0.056	0.015	[.029,.088]

The correlations between five aspects of school climate and teachers’ enjoyment and self-efficacy were examined, and the results revealed that teachers’ perceptions of collaboration with colleagues, school resources, instructional innovation, and involvement in decision-making were directly and positively related to teacher enjoyment (supporting H1.1, 1.3, 1.4, 1.5). However, the path between teacher-student relationships and teacher enjoyment (H1.2) was not statistically significant (*β* = −0.008, *p* = 0.854). In terms of teacher self-efficacy, the results revealed that a collaborative and innovative school climate were significant predictors of teachers’ self-efficacy (supporting H2.1, H2.4). Conversely, the paths between teacher-student relationships, school resources, participation in decision-making, and teachers’ self-efficacy (H2.2, H2.3, H2.5) were not significant (*β* = −0.039, *p* = 0.384; *β* = 0.084, *p* = 0.051; *β* = 0.071, *p* = 0.119).

Additionally, through the bootstrapping method with 5000 re-samplings, we examined the significance of indirect effects (see Table 4). Our results confirmed that teacher self-efficacy functions as a mediator between two relationships. Specifically, instructional innovation and collaboration with colleagues exerted significant indirect effects on teacher enjoyment via teacher self-efficacy (supporting H3.1, H3.4).

## Findings and discussion

This study contributes to the existing body of knowledge on the mechanisms underlying the relationships between school climate factors, teacher enjoyment, and self-efficacy. Three key findings emerge from the result analysis. First, four of the five school climate-collaboration with colleague collaboration, decision-making involvement, resource availability, and instructional innovation-demonstrated significant positive effects on teacher enjoyment. Second, instructional innovation and collaboration in the workplace showed direct positive associations with teacher self-efficacy. Furthermore, mediation analysis confirmed teacher self-efficacy’s pivotal role in mediating the effects of these two factors on teacher enjoyment.

### School climate and teacher enjoyment

The study findings demonstrated that school climate factors significantly influence teachers’ emotional experiences, with positive perceptions of the work environment correlating with increased teacher enjoyment. This confirms that teacher enjoyment is not merely an individual psychological state, but an eco-psychological construct shaped by ongoing teacher-environment interactions. This co-constructive perspective corresponds with Bronfenbrenner’s theory, which emphasized that human development arises through continuous, reciprocal interactions with ecological systems [94]. Building upon this framework, we explored the role of various school climate factors in shaping teacher enjoyment at both the micro- and mesosystem levels.

At the micro level, teachers’ perception of collaboration with colleagues is positively associated with their enjoyment, indicating that collegial interactions, such as co-teaching, joint lesson planning, collaborative curriculum development, and discussions regarding students’ individual needs, contribute to more positive emotional outcomes. This finding not only aligns with Erb’s observation regarding colleague-supported positive emotions but also quantitatively corroborates Huang et al.’s established association between collaboration and positive emotions [95,96]. Specifically, when teachers opt to work together, the social interaction inherent in teamwork serves as a key mechanism for enhancing their work enjoyment [97,98]. In theory, this result validates Fredrickson’s Broaden-and-Build Theory explaining that effective collaboration provides both material and emotional support, helping individuals build personal resources that can increase joy, achievement, and a sense of community [99–101]. However, our study found no direct correlation between teacher-student relationships and teacher enjoyment among Chinese college teachers. This could be attributed to the complex nature of teacher-student relationships, which are deeply shaped by cultural and community contexts [102]. In Chinese culture, traditionally influenced by Confucian values, there is a strong emphasis on hierarchy relationships [103], where teachers are seen as authoritative figures whose actions are rarely questioned by students [104]. The respect fosters cooperation, which in turn facilitates classroom teaching [105]. However, this respect and cooperation are often based more on cultural expectations and formality rather than on emotional or interpersonal dynamics. As such, while these cultural norms create a harmonious classroom environment, they may also lead to a more formalized and less emotionally engaging teacher-student relationship. This lack of emotional connection may, in turn, limit the extent to which teacher-student interactions influence teachers’ enjoyment of their work.

At the meso level, the availability of school resources plays a crucial role in fostering teachers’ positive affect [106]. Previous studies showed that a lack of support and resources from school administrators can exacerbate teachers’ negative emotions and stress, leading to burnout and compromising their well-being [107]. In contrast, our findings suggested that access to abundant educational materials enhances teachers’ positive emotions. This finding partially supports Collie et al.’s study, which emphasized the significance of contextual factors—such as adequate facilities, equipment, and resources—in shaping teachers’ emotional experiences and well-being [4]. Additionally, instructional innovation emerges as an important factor influencing teacher enjoyment—an area that has not been sufficiently explored in previous research. While existing studies have not explicitly examined this direct link, two established mechanisms have provided plausible theoretical support: First, teachers’ innovative teaching practices have been shown to increase student enjoyment [108,109]. Second, research established a clear emotional transmission pathway between student enjoyment and teacher enjoyment in the classroom [11,26,110]. Although these mechanisms have emphasized an indirect pathway, our results suggested that instructional innovation may also exert a direct emotional impact on teachers, potentially by fostering a sense of autonomy, competence, or fulfillment in the creative process itself. Furthermore, our data revealed that teachers’ involvement in school decision-making, particularly in curriculum development and policy formation, is a major indicator of teachers’ enjoyment. This effect occurs because meaningful involvement boosts teachers’ sense of worth, empowerment, and self-confidence [111]. This aligns with Huang et al.’s empirical evidence, which established a connection between teachers’ engagement in institutional decision-making and their positive emotional well-being [96].

### School climate and teacher self-efficacy

The following discussion examines how teacher self-efficacy is influenced by school climate factors operating across multiple ecological levels. At the micro level, this study reaffirmed the significant role of colleague collaboration in enhancing teachers’ self-efficacy, a relationship well-documented in previous research [4,19,112]. This finding aligns with the Teaching and Learning International Survey (TALIS) 2013, which identified school collaborative culture as one of the most significant predictors of both teacher self-efficacy and job satisfaction [113]. The mechanism underlying this effect operates through two primary pathways: First, collaboration provides teachers with chances for reflection, allowing them to thoroughly examine and address issues in their lessons [114]. Second, through such interactions, teachers gain the confidence to experiment with new and different instructional strategies in their classes [115]; Even for educators who are not actively seeking innovation, collaborating with peers can boost their confidence and self-efficacy [116]. Second, while prior studies demonstrated that positive teacher-student relationships can significantly enhance teachers’ self-efficacy through reciprocal dynamics [4,117,118], the results of this research revealed a different scenario in Chinese college settings. Specifically, students’ active participation in lessons or well-mannered and respectful attitudes do not appear to directly impact how college teachers perceive their self-efficacy. As explained above, this discrepancy may be attributed to the greater emphasis on respect for authority and hierarchical relationships between teachers and students in Chinese educational contexts.

At the meso level, instructional innovation emerges as a critical factor in strengthening teachers’ self-efficacy. This finding supports a thread of research findings highlighting how incorporating innovative teaching methods and fostering a supportive teacher-learning environment elevate teachers’ confidence and faith in their capabilities [20,48,83]. The results emphasized the importance of fostering a supportive school climate where teachers can try innovative teaching approaches and diverse ideas, and where teachers are also given ongoing opportunities for implementing new courses or curriculum materials, leading to the gradual enhancement of instructors’ self-assurance in instructional strategies, student engagement, and classroom management. Second, results also showed that teachers’ participation in school decision-making—another important aspect of school climate—is not correlated with teachers’ self-efficacy. This echoes Bandura’s argument that empowerment is ineffective unless individuals develop internal efficacy to utilize opportunities [119]. However, empirical studies have presented mixed findings: while Brown found negative correlations between self-efficacy and teachers’ perception of their control over work situations [120], Hemric et al. presented a contrasting viewpoint, arguing teachers who are given autonomy in their roles exhibit greater self-efficacy when compared to those who have very little say in the school policy and development [121]. Furthermore, two global research reviews outlined research findings that convincingly demonstrate the positive impact of teacher autonomy on their self-perceived self-efficacy [122,123]. Therefore, variations exist in teachers’ perceptions of decision-making and self-efficacy. Consequently, further investigation with a larger sample in a similar setting may be necessary to validate this relationship. Lastly, this research failed to confirm the significant relationship between resource adequacy in schools and teachers’ self-efficacy, as evidenced in earlier studies [19,51,52]. Therefore, additional exploration with more extensive samples in a comparable environment could be vital to confirm or refute these relationships.

### Mediating roles of teacher self-efficacy

In addition to identifying the distinct connections between various aspects of school climate and teacher self-efficacy, this study highlighted the critical mediating role of teacher self-efficacy. To note, we focused on mediation given previous studies supporting school climate’s indirect effect on teacher enjoyment through self-efficacy. While moderation (e.g., self-efficacy as a buffer) was beyond the scope of this study, it warrants further investigation.

First, the findings clearly indicated that in schools characterized by a supportive environment for innovation, teachers’ self-efficacy is likely to increase, leading to greater joy in their professional lives [124]. Specifically, at the microsystem level, when teachers engage in productive professional coordination and communication, they build stronger confidence in their instructional capabilities [4,19,112], which in turn enhances their overall teaching enjoyment. Additionally, the implementation of innovative teaching methodologies, when supported by strong teacher self-efficacy, can significantly enhance teacher enjoyment at the mesosystem level. This finding aligns with Pekrun’s control-value theory of achievement emotions, which posited that teachers who possess a growth mindset perceive their teaching ability as an aspect they can influence and enhance through dedicated effort, leading to higher control appraisals and, consequently, higher levels of positive emotions contrasted with individuals holding a fixed mindset [125].

## Conclusion and implications

Teachers’ enjoyment and self-efficacy deserve attention from researchers, administrators, and policymakers as well, especially considering teachers’ high attrition and turnover rates worldwide [126,127]. Drawing on Bronfenbrenner’s ecological systems theory, this study examined the relationships between environmental factors (i.e., school climate) and personal factors (i.e., enjoyment and self-efficacy) among college teachers. The results first uncovered how various school climate factors contributed to teachers’ variance in enjoyment and self-efficacy. Specifically, teacher collaboration and instructional innovation were confirmed to positively influence teacher self-efficacy, while collaboration, school resources, instructional innovation, and decision-making were linked to increased teacher enjoyment. The results further highlighted the mediating role of self-efficacy in the relationship between innovative and collaborative school climate and teacher enjoyment. Theoretically, the study enriches our understanding of the complex interplay between contextual and psychological factors. Practically, the findings offer valuable insights for educators, school leaders, and policymakers within China specifically and more broadly in all educational contexts.

First, it is crucial for colleges to foster a school climate centered on collaboration, where staff members help and support one another, in that collaborative activities that facilitate opportunities for teachers to work together and support one another can help enhance both teacher self-efficacy and enjoyment. Mentorship programs can be created by schools that match experienced educators with novice teachers, offering them guidance, support, and insights, helping new teachers navigate challenges and grow in their roles. In such a school environment, it is essential for teachers to be inspired to be innovative in instruction. Teachers should be granted the freedom and autonomy by school leaders to make decisions regarding their classrooms and teaching methods. Empowering them to experiment with new teaching methods, curriculum design, and assessment strategies, allows for creativity and innovation. Workshops, seminars, and courses focused on instructional strategies can be arranged to assist teachers in recognizing and appreciating their achievements, thereby enhancing their self-efficacy and sense of competence. Moreover, ample resources are essential for increasing teacher enjoyment. Schools should ensure teachers have access to comprehensive curriculum resources, necessary materials and supplies, technology infrastructure, and educational technology tools. Administrators should also grant teachers appropriate decision-making rights. Establishing regular platforms for communication and feedback enables teachers to express their needs and concerns. Additionally, professional development workshops can incorporate mindfulness interventions and emotion regulation techniques [128,129] to support teachers in cultivating positive emotions and equip them with practical strategies to handle challenges and setbacks and further strengthen their sense of efficacy.

## Limitations

To start, this study used self-reported questionnaire data to test the proposed model, a method commonly used to evaluate teachers’ emotional experiences [130]. Nevertheless, it is vital to acknowledge that this approach may have potentially strengthened the relationships among the variables under investigation. As noted by Winograd, teachers may be inclined to overstate their enjoyment when reflecting on their emotional experiences in the classroom, possibly due to societal expectations that teachers should always find joy in their work [131]. Additionally, the quantitative design of the study limited the opportunity to capture participants’ personal voices and unique experiences. Therefore, further research employing mixed-methods approaches and incorporating data from multiple sources such as students, classroom observers, and administrators is necessary to triangulate data and deepen our understanding of the connections between the key variables in this research. Second, while snowball sampling enabled efficient recruitment of teachers, its self-selection nature may bias the sample toward individuals with stronger social connections or shared perspectives. Future studies could benefit from adopting hybrid sampling strategies to ensure more diverse representations and enhance the validity of the findings. Third, given that this study was carried out on Chinese college teachers whose experiences and workplace environments may be different from those of teachers in other contexts and cultural backgrounds, it is essential for future research to include a broader range of samples to ensure the generalizability of the findings. For example, studies could examine teachers across different educational levels (e.g., elementary or secondary school), or in non-Asian cultural contexts to assess whether the observed relationships hold across diverse educational settings. Finally, in this study, factors like teachers’ career stages or age groups were not taken into account in the model. As the career stage has been shown to influence teachers’ psychological experiences [18,132], future studies are recommended to explore how career stages influence the relationships among the main variables in this study.

## Supporting information

S1 Data https://doi.org/10.6084/m9.figshare.29371211.v1. (XLSX)
